# Clinical analysis of AN69ST membrane continuous venous hemofiltration in the treatment of severe sepsis

**DOI:** 10.1515/med-2023-0784

**Published:** 2023-09-12

**Authors:** Yuqiang Gao, Xiaohong Huang, Yanan Yang, Zhenlin Lei, Qingan Chen, Xu Guo, Jia Tian, Xiaoxin Gao

**Affiliations:** Intensive Medical Unit, Hainan Medical University, 571199 Haikou, China; Intensive Medical Unit, Hainan General Hospital, Hainan Affiliated Hospital of Hainan Medical University, 570311, Haikou, China; Intensive Medical Unit, Hainan General Hospital, Hainan Affiliated Hospital of Hainan Medical University, No. 19 Xiuhua Road, 570311 Haikou, China

**Keywords:** sepsis, cytokine adsorption, AN69ST, blood purification, clinical efficacy

## Abstract

We aimed to investigate the clinical efficacy of blood purification technology based on cytokine adsorption in the treatment of sepsis. Sixty patients with sepsis were randomly divided into control (*n* = 30) and experimental (*n* = 30) groups. Both groups were given routine treatment and continuous venovenous hemofiltration, and on this basis, the experimental group received acrylonitrile/sodium methacrylate (AN69ST) blood purification. The levels of C-reactive protein, procalcitonin, white blood cell count, albumin, platelets, total bilirubin, creatinine, lactic acid, and APACE II score, as well as secretion of inflammatory factors interleukin (IL)-6 and tumor necrosis factor (TNF-α) were compared. The hospitalization time, mechanical ventilation (MV) time, drug use time, and mortality were analyzed. After treatment, the secretion levels of IL-6 and TNF-α were decreased, and other indicators were significantly improved compared with those before treatment (*P* < 0.05), especially in the experimental group (*P* < 0.05). The hospitalization time, MV time, and drug use time in the experimental group were significantly lower than those of the control group (*P* < 0.05), and the mortality was lower than that in the control group (*P* < 0.05). In conclusion, blood purification technology based on cytokine adsorption can significantly improve various indicators of sepsis patients, reduce hospitalization time, reduce mortality, and improve the prognosis.

## 1 Introduction

Sepsis and septic shock are serious inflammatory diseases associated with high incidence rate and mortality [1]. The innate immune system is the first line of defense against bacterial infection [2]. Inflammation caused by severe sepsis can lead to organ failure or circulatory dysfunction in varying degrees. There are 20–30 million patients suffering from sepsis every year in the world. According to the literature, the number of sepsis cases is increasing by 1.5–1.8% every year. The case fatality rate of this disease can reach 30–45%. Sepsis has become an important cause of death in ICU patients, which is posing a serious threat to human health [3]. The common pathogens causing sepsis mainly include staphylococcus, streptococcus, enterococcus, gram-negative bacilli, and fungi [4]. Sepsis is now defined as the maladjustment of host’s response to infection, leading to life-threatening organ dysfunction. Moreover, it is related to the strong stimulation of pathogen-associated molecular patterns (PAMPs) and damage-associated molecular patterns (DAMPs) on pattern recognition receptors (PRRs), which leads to the production of pro-inflammatory cytokines, such as tumor necrosis factor (TNF)-α, interleukin (IL)-1β, IL-6, and IL-8 [5]. Subsequent biological reactions are believed to develop into diseases at the molecular and cellular level, such as cell death, microcirculation disorders, and mitochondrial dysfunction, and diseases at the organ level, such as lung injury, sepsis-related acute kidney injury (AKI), septic cardiac insufficiency, and sepsis-related encephalopathy [6–11]. Continuous venovenous hemofiltration (CVVH) is a continuous renal replacement therapy (CRRT) technology, which can provide additional advantages in the treatment of ICU patients by removing proteins of medium molecular size (such as cytokines) [12,13]. CVVH uses a large amount of displacement fluid instead of dialysate, so the convection effect of solute removal completely depends on the ultrafiltration rates (UFR). However, there is limited evidence that high-volume hemofiltration can improve the prognosis of critically ill patients [14,15]. On the other hand, the filtration membrane of acrylonitrile/sodium methacrylsulfonate (AN69ST) has shown its effect on the expression of cytokines (TNF-α, IL-1β, IL-6, IL-8, and IL-10) and high mobility group box-1 (HMGB1), which are representative of damage-related molecular models in experimental research [16–18].

The first step of the infectious process is the recognition of the pathogen by the immune system. All pathogens exhibit on their surface specific components, known as PAMPs, such as the endotoxins expressed by Gram-negative bacteria. During infection, PAMPs are recognized by the PRR expressed at the surface of immune cells [4]. This signal activates the leukocytes and induces the synthesis of pro- and anti-inflammatory cytokines, including TNF-α, IL-1, IL-6, IL-8, and IL-10. The massive release of cytokines in the blood has been described as a “cytokine storm” and is believed to be responsible for major organ dysfunctions [5,6].

Injured host cells express on their surface DAMPs, such as the HMGB1 protein. DAMPs may be released in the circulation and are recognized by the PRR, thus enhancing leukocyte activation and cytokine synthesis, fuelling the vicious circle of uncontrolled immunoinflammatory process [7]. After the initial cytokine storm, an immunoparalysis state occurs, contributing to most of the sepsis-associated deaths because of health-care-associated infections and viral reactivations [8].

Addressing the unbalanced immune answer to infection has been a therapeutic challenge for many years.

However, a better understanding of the mechanisms underlying sepsis has permitted to develop new immune therapies to modulate the inflammatory process. Promising results have been obtained with new molecules such as recombinant human IL-7 [9]. Another approach consists of removing a nonspecific broad spectrum of inflammatory mediators. This is now possible, thanks to the industrial advances and the development of extracorporeal blood purification devices [10]. Most of these extracorporeal techniques interfere at one particular step of the complex immune process, but some of them may have two or more targets. Various hypotheses have been developed to explain their effects. First, they may decrease cytokine concentrations under a “toxic threshold” in order to limit the local deleterious effects of cytokines [5]. Other authors have hypothesized that because of a restored concentration gradient, the decrease in cytokine blood concentrations could promote leukocyte chemotaxis toward infected tissue where cytokine concentrations are higher [11]. Another target of the blood purification techniques is the inhibition of the immunoinflammatory cascade trigger. The objective is therefore to remove pathogens or PAMPs such as endotoxins before they activate leukocytes [12]. Finally, the modulation of the immune process may directly involve the leukocytes, either through their direct removal or through an immune cell reprograming (modulation of surface markers expression, improvement of antigen-presenting capability, or adjustment of apoptosis) [13,14].

A particular surface treatment was added to the native AN69 membrane. The surface treatment consists of a coating with polyethyleneimine (PEI), a positively charged molecule that allows for a better biocompatibility by reducing the zeta potential of the membrane and thus the bradykinin production. The PEI coating also offers anti-thrombogenic opportunities as the hemofilter may be primed with a heparinized solution (the free positive charges of the cationic PEI polymer are able to adsorb the negatively charged heparin molecules); the adsorbed heparin is fixed on the membrane surface but remains active. Prospective studies reported successful reduction of systemic heparin dose for chronic intermittent hemodialysis in patients at high risk of bleeding when using a heparin-primed AN69 surface-treated (AN69ST) membrane.

The second advantage of the AN69ST is that its capacity to remove cytokines is preserved despite the surface treatment. For instance, Yumoto et al. [19] reported the results of an *in vitro* comparison among four different hemofilters for the removal of HMGB1, a key mediator of sepsis-induced inflammation. In this study, the AN69ST membrane exhibited better HMGB1 removal as compared to PMMA membrane and much better removal than polyarylethersulfone and PS membranes [19]. The adsorptive capacities of the AN69ST were also clinically confirmed in acute patients treated with CRRT and an AN69ST membrane.

AN69ST hemofilter, also known as cytokine adsorption blood filter, is known to adsorb inflammatory mediators [20–24]. At present, AN69ST membrane has attracted attention in the field of intensive care because of its ability to adsorb cytokines, which makes it potentially beneficial in the treatment of patients with septic shock [25,26]. In addition, in Japan, continuous hemo(dia)filtration (CH(D)F) is not only used to replace renal function, but also used to prevent or treat organ damage by removing inflammatory mediators [27]. Given that sepsis is the most common cause of AKI in the ICU and is a strong predictor of in-hospital mortality, it is critical to evaluate the impact of CVVH and AN69ST filters, which have a high absorption capacity on sepsis-induced mortality in patients. However, few experiments have investigated the clinical efficacy of AN69ST membrane blood purification on severe sepsis; therefore, in this study, we applied AN69ST membrane adsorption and CVVH technology to treat severe sepsis patients and evaluated its clinical efficacy.

## 2 Methods

### 2.1 General information

A total of 60 patients who were admitted to the Department of critical medicine of Hainan Provincial People’s Hospital for the treatment of severe sepsis between January 2020 and October 2020 were selected for the study. Among them, 30 were in the experimental group and 30 were in the control group. Inclusion criteria: (1) the admission fulfilled the diagnostic criteria of sepsis according to the 2016 international management guidelines for severe sepsis and septic shock [3]. Exclusion criteria: (1) patients who did not meet severe sepsis, (2) received renal replacement therapy (RRT) modality other than CVVH or a hemofilter other than AN69ST, (3) patients who received RRT before admission, spent less than 48 h in hospital or had missing laboratory data between admission and 24 h from the start of CVVH, and (4) patients with COVID. Finally, 60 patients were included, and the patients were equally divided into experimental and control groups using the random number method, with 30 patients in each group. Eligible participants were followed up until their death or hospital discharge.


**Ethical approval and consent to participate:** This study was approved by the Ethics Committee of Hainan General Hospital, Hainan Affiliated Hospital of Hainan Medical University (20201023) and written informed consent was obtained from the patient.

### 2.2 Data collection

Clinical data such as patients’ gender and age as well as chronic underlying diseases were collected from each group. The control group consisted of 16 males and 14 females; age range 28–76 years, mean (46.78 ± 18.16) years; comorbid chronic underlying diseases hypertension (*n* = 11), diabetes mellitus (DM) (*n* = 9), coronary heart disease (*n* = 8), chronic cardiac insufficiency (*n* = 7), chronic obstructive pulmonary disease (COPD) (*n* = 10), and chronic renal insufficiency (*n* = 19). Mean body mass index (BMI) (23.6 ± 2.11) kg/m^2^; and evaluation of acute physiology and chronic health evaluation (APACHE II score), mean APACHE II (16.59 ± 1.70) points. There were 18 males and 12 females in the experimental group; age ranged from 27 to 78 years (mean, 47.25 ± 16.83). Concomitant chronic underlying diseases hypertension (*n* = 13), diabetes (*n* = 10), coronary heart disease (*n* = 6), chronic cardiac insufficiency (*n* = 8), COPD (*n* = 7), and chronic renal insufficiency (*n* = 22). Mean BMI (23.64 ± 2.13) kg/m^2^; mean acute physiology and chronic health evaluation system II (APACHE II) scores (16.56 ± 1.69). There was no significant difference in the general data between the two groups (*P* > 0.05).

### 2.3 Therapeutics

The control group as well as the experimental group underwent routine comprehensive treatment. That is, all patients were treated with active anti-infective treatment, symptomatic treatment of primary disease, correction of water electrolyte balance and acid–base balance, nutritional support, and if necessary, patients were intubated, mechanically ventilated, or indwelling deep veins. Its anti-infective treatment needs to be based on the patient’s clinical manifestations, G-test and GM test, and imaging findings, with a reasonable choice of antibiotics to ensure that the antibiotic will cover the treating pathogenic microorganism. Then we choose the right antibiotic on the basis of blood culture, drug susceptibility test results. Invasive ventilator-assisted ventilation, high flow oxygen therapy was given when the respiratory oxygenation index was <200. When heart failure and AKI are present, extracorporeal membrane oxygenation (ECMO) fluid management, bedside CRRT replacement, appropriate sedation and analgesia, and nutrition support therapy were given. Control group adsorptive blood purification (sepxiris100; Baxter Co. Ltd, Tokyo, Japan) with PMMA membrane (Hemofeel CH1.0N; Toray Medical Co., Ltd) on the basis of routine comprehensive treatment. Control group received adsorptive blood purification (sepxiris100; Baxter Co. Ltd, Tokyo, Japan) with PMMA membrane (Hemofeel CH1.0N; Toray Medical Co., Ltd) on the basis of routine comprehensive treatment. Experimental group received adsorptive blood purification (sepxiris100; Baxter Co. Ltd, Tokyo, Japan) with acrylonitrile/sodium methacrylate (AN69ST) membrane on the basis of routine comprehensive treatment. The device used in this study was jun-505 (Junken medical, Co., Ltd, Tokyo, Japan). Heparin coating was performed by adding sodium heparin to the filling solution, and the patient was treated continuously for 3 days, after stable disease and significant improvement in various biochemical parameters and symptoms, the treatment could be stopped. Treatment was given as one session over 1 week for a total of three sessions.

### 2.4 IL-6 and TNF-α measurement

The levels of IL-6 and TNF-α were measured by the commercial reagent kits (Nanjing Jiancheng Bioengineering Research Institute, Nanjing, China).

### 2.5 Outcome measures

We obtained basic information of the participants on the first day of hospitalization, including demographic data (age, sex, baseline renal function, weight, and height), medical or surgical hospitalizations, major infectious source of sepsis, and comorbidities. Etiologies of sepsis include pulmonary infections, urinary tract infections, intra-abdominal infections, soft tissue infections, central nervous system infections, and bloodstream infections. Comorbidities include DM, hypertension, cirrhosis, coronary artery disease, congestive heart failure, COPD, cerebrovascular disease, chronic kidney disease, and malignancy. In addition, we recorded details of the medical interventions performed by the participants during their ICU stay, such as the need for diuretics and vasoactive drug support (vasopressors and inotropic agents), the use of mechanical ventilation (MV), intra-aortic balloon pumps, and ECMO. Recorded parameters were blood flow settings for UFR and CVVH, and laboratory data according to cumulative fluid balance, mean arterial pressure (map), ratio of partial pressure of arterial oxygen to fraction of inspired oxygen (PaO_2_/FiO_2_), and 24 h after initiation of CVP and CVVH were collected. On the first day of ICU admission and on the day CVVH was started, we calculated the APACHE II score to estimate the severity of the disease. In addition, we recorded levels of C-reactive protein (CRP), procalcitonin (PCT), white blood cell count (WBC), albumin (ALB), platelet (PLT), total bilirubin (TBIL), creatinine (CR), lactic acid (LAC), as well as inflammatory cytokines IL-6 and TNF-α secretion profile, and analyzed the length of hospital stay, duration of MV, medication use, and 30-day case fatality. Sepsis is a multiple organ dysfunction syndrome (MODS) caused by the host’s excessive stress on infection and immune imbalance, and also an important cause of death for critically ill patients. The mortality rate of sepsis is very high, reaching 24.3%, which seriously threatens the life safety of patients. Sepsis myocarditis is one of the most common complications of sepsis. It is also the first cause of death of sepsis patients. According to the report of Bouhelmod et al., the incidence rate of septic myocarditis in sepsis patients is close to 20%.

### 2.6 Statistical analysis

SPSS 26.0 software was used for data statistical analysis. Count data were expressed as percent, and differences between groups were compared using *χ* ^2^ test; measurements that conformed to normal distribution were presented as (*x* ± *s*), and differences between groups were compared by two independent samples *t*-test. *P* < 0.05 represents a significant difference.

## 3 Result

### 3.1 Comparison of patients’ clinical baseline characteristics

Patient clinical baseline characteristics were compared between the control and experimental groups, respectively. There were no significant differences in the general data including gender, age, or chronic underlying diseases between the two groups (*P* > 0.05), which were comparable and is as shown in Table 1.

**Table 1:** Clinical baseline characteristics of patients

Item	Experimental group	Control group
Age (years)	47.25 ± 16.83	46.78 ± 18.16
Gender (male/female)	18/12	16/14
**Chronic basic diseases**		
Hypertension	13	11
Diabetes	10	9
Coronary heart disease	6	8
Chronic heart failure	8	7
COPD	7	10
Chronic renal failure	22	19

### 3.2 Improvement of clinical inflammatory indexes

Clinical inflammatory parameters were analyzed before and 1 week after treatment in both the groups. We analyzed CRP, PCT, WBC, respectively. The results showed that before treatment, there was no significant difference in the clinical indicators of inflammation between the two groups (*P* > 0.05), whereas after treatment, the clinical indicators of inflammation in the two groups were significantly reduced (*P* < 0.05), and the experimental group improved better than the control group (*P* < 0.05) (Table 2).

**Table 2:** Clinical inflammatory indexes in patients with sepsis

Group	CRP (mg/L)		PCT (ng/mL)		WBC (×10^9^)	
	Before treatment	After treatment	Before treatment	After treatment	Before treatment	After treatment
Control group	82.37 ± 7.86	45.05 ± 5.29*	6.93 ± 1.21	3.03 ± 0.89*	14.08 ± 2.31	10.50 ± 2.13*
Experimental group	79.85 ± 9.35	32.56 ± 4.38*^#^	7.19 ± 1.37	1.98 ± 0.78*^#^	16.18 ± 2.89	7.13 ± 2.78*^#^

Notes: compared with the same group before treatment, *P＜0.05; compared with the control group after treatment, ^#^ *P*＜0.05.

### 3.3 Analysis of inflammatory factor influence

The inflammatory cytokines IL-6 and TNF-α secretion profile were further analyzed in both patients before and 1 week after treatment. The results showed that before treatment, there was no significant difference in the levels of inflammatory factors between the two groups (*P* > 0.05), whereas after treatment, the secretion levels of inflammatory factors in the two groups were significantly reduced (*P* < 0.05), and the improvement in the experimental group was better than that in the control group (*P* < 0.05) (Table 3).

**Table 3:** Inflammatory factors in patients with sepsis

Group	IL-6 (pg/mL)		TNF-α (pg/mL)	
	Before treatment	After treatment	Before treatment	After treatment
Control group	432.59 ± 35.61	192.71 ± 21.34*	236.93 ± 22.51	143.07 ± 17.81*
Experimental group	459.66 ± 41.75	112.54 ± 14.67*^#^	267.19 ± 31.49	116.32 ± 11.56*^#^

Notes: compared with the same group before treatment, *P＜0.05; compared with the control group after treatment, ^#^ *P*＜0.05.

### 3.4 Analysis of hepatic and renal effects

Liver and kidney function measurements were analyzed before and 1 week after treatment in both the groups. The results showed that before treatment, there were no significant differences between the two groups in the liver and kidney function including ALB, PLT, TBIL, CR, and LAC (*P* > 0.05). However, after treatment, both patients improved significantly (*P* < 0.05) in the above indicators, and the experimental group improved better than the control group (*P* < 0.05) (Table 4).

**Table 4:** Changes of liver and kidney function in patients with sepsis

Group	ALB (g/L)		PLT (g/L）		TBIL (μmol/L)		Cr (μmol/L)		LAC (mmol/L)	
	Before treatment	After treatment	Before treatment	After treatment	Before treatment	After treatment	Before treatment	After treatment	Before treatment	After treatment
Control group	21.47 ± 5.75	32.19 ± 7.33*	415.67 ± 68.81	323.67 ± 32.17*	24.57 ± 7.25	17.54 ± 4.42*	329.74 ± 62.69	152.61 ± 35.41*	3.75 ± 0.67	2.34 ± 0.56*
Experimental group	20.85 ± 8.18	39.49 ± 8.41*^#^	437.68 ± 72.58	298.53 ± 24.22*^#^	26.63 ± 6.83	15.22 ± 3.79*^#^	341.89 ± 62.69	135.23 ± 28.41*^#^	3.95 ± 0.79	1.74 ± 0.78*^#^

Notes: compared with the same group before treatment, *P＜0.05; compared with the control group after treatment, ^#^ *P*＜0.05.

### 3.5 Analysis of length of hospital stay, duration of MV, and medication use

The results showed that patients in the experimental group had a significant reduction (*P* < 0.05) in hospital stay, duration of MV, and duration of medication use when compared with the control group (Table 5).

**Table 5:** Hospitalization time, MV time, and drug use in patients with sepsis

Item	Control group	Experimental group
Hospitalization time (days)	21.57 ± 7.38	16.26 ± 7.21*
MV time (days)	18.03 ± 5.11	15.40 ± 4.39*
Vasoactive drugs (days)	4.94 ± 2.12	3.37 ± 1.53*
Antibiotic (days)	19.33 ± 3.57	14.09 ± 2.18*

Note: compared with the control group, *P＜0.05.

### 3.6 APACE II score and analysis of 30-day case fatality

We finally analyzed the prognostic impact of the sixty-ninth membrane adsorbed blood purification in patients with severe sepsis to analyze the APACE Ⅱ score as well as the 30-day case fatality rate, respectively. The results showed that, before treatment, there was no significant difference in APACE II scores between the two groups (*P* > 0.05). After treatment, the two groups of patients were significantly improved (*P* < 0.05). However, compared with the control group, the APACE II score of patients in the experimental group was significantly improved (*P* < 0.05).

## 4 Discussion

Sepsis is a multiple organ dysfunction disease. Sepsis is defined as “a life-threatening organ dysfunction caused by a dysregulated host response to infection” [1]. “Dysregulated host response” indicates that the cytokine profile varies between individuals in sepsis. Imbalances of pro- and anti-inflammatory cytokines may lead to MODS [2]. Pro-inflammatory cytokines, including IL-1β, IL-6, IL-12, and TNF-α, are released by host cells in response to DAMPs and PAMPs. Anti-inflammatory cytokines including IL-1 receptor antagonist, IL-4, and IL-10 also play an important part to reduce the overall production of pro-inflammatory cytokines. However, overexpression of the anti-inflammatory cytokines can also lead to immunoparalysis following sepsis [3] and high risk of secondary infection. Its pathogenesis involves different aspects such as inflammatory effect, immune dysfunction, tissue damage, coagulation function, and host’s abnormal response to different infectious pathogens. The complications of sepsis may include septic shock, MODS, and a series of pathological changes, such as persistent low-level inflammation, catabolism, and immune paralysis [28,29]. Severe sepsis is a serious stage of sepsis, which refers to the occurrence of one or more organ dysfunction on the basis of sepsis, of which renal failure is one of its symptoms [30]. In the human body, the kidney is responsible for excreting waste, maintaining the acid–base balance of the body, regulating ion balance and other functions. Renal failure will lead to the inability to remove toxins in the blood and the imbalance of electrolytes in the body. Therefore, CH(D)F is applied to the treatment of sepsis patients. In recent years, CH(D)F has been widely used in emergency treatment of common critical diseases, which become one of the most important support measures for treating various critical diseases. Adsorption is expected to be used to remove media with large molecular weight, such as cytokines. Although the adsorbent is usually designed as diheptyl phthalate column, CH(D)F is easier to use in ICU which can achieve cytokine removal and kidney support treatment. CH(D)F is internationally known as “renal (renal) replacement therapy” and has been used outside Japan for AKI [31]. But there are few studies targeting the removal mediators of sepsis. Research data suggests that cytokine adsorption filters with or without novel immunomodulatory columns can be applied to the treatment of sepsis [32].

MiRNA is an endogenous non-coding RNA molecule composed of 19–25 nucleotides, which is synthesized in the cytoplasm by RNA polymerase 2 in the nucleus acting on gene transcription and post-transcriptional processing modification, and enters the circulation mainly in the form of microbubbles [9]. Among sepsis patients, the incidence of myocardial injury is high, and the mortality rate is as high as 70–90% [10]. Sepsis-related myocardial injury will cause damage to myocardial cells, which will lead to limited cardiac systolic function and decreased ejection fraction. Its pathogenesis is complex, involving inflammatory factors, oxidative stress, etc. [11]. It was found that MiRNA-125b could target TRAF6-mediated NF-κ B pathway, significantly inhibit CAM-1 expression, resist the aggregation of macrophages and neutrophils in myocardial tissue, and reduce serum TNF-α and IL-1β horizontal [12]. Wang et al. reported that MiRNA-27a can reduce NF-κ phosphorylation level of Bp65 protein, inhibition of its DNA binding activity, and regulation of peroxisome proliferator activated receptor γ level, and downregulation of TNF-α to reduce the inflammatory reaction of septic rats [13]. MiR-133a can reduce heart damage caused by sepsis by targeting BNIP3L [14]. Long chain non-coding microRNA-133a-3p upregulates aquaporin 1 to reduce LPS-induced inflammatory response in sepsis [15]. MiR-133a originates from the myocardium and has the specificity of striated muscle tissue. It participates in the normal growth and development of the heart and is differentially expressed in the pathophysiological processes such as myocardial cell hypertrophy and ventricular remodeling, so it is closely related to myocardial cell damage [16].

Since Parker and others first reported the reversible myocardial dysfunction in sepsis patients in 1984, scholars have continuously deepened their research on septic myocarditis; especially in recent years, with the popularization of bedside echocardiography and other examinations, people have gained a deeper understanding of septic myocarditis [3]. However, at present, the pathogenesis of septic myocarditis is still not fully clarified, and the research on its treatment is also slow. Therefore, it is of great clinical significance to carry out research on the treatment of septic myocarditis.

In the early 1990s, Bellomo et al. stated that CRRT treatment slowed the progression of AKI and removed inflammatory cytokines from the circulation of septic patients [33]. Servillo et al. reported that the immunomodulatory effects of CVVH could be obtained using a high UFR of 60 mL/kg/h in critically ill patients with AKI associated with severe sepsis or septic shock [12]. Although the average cut-off value of modern high-throughput membrane is about 30–40 kD, it should be able to eliminate inflammatory cytokines through convection. However, considering the high production rate and turnover rate of media, some people doubt whether the amount removed has clinical significance [12]. In addition, De Vriese et al. demonstrated that CVVH removes cytokines from the circulation of sepsis patients by adsorption within the first hour after putting a new hemodialyzer into the circuit [34]. However, the filter membrane is rapidly saturated with cytokines, and the anti-inflammatory medium is removed to the same extent as inflammatory cytokines, which may explain the lack of survival benefits of high-intensity CRRT in previous studies [35,36]. Moreover, Lee’s results showed that sepsis patients who received AN69 membrane treatment with a higher UFR in CVVH had improved 90-day survival [36]. The AN69 membrane is a copolymer of acrylonitrile and sodium methylallylsulfonate and is characterized by a strong negative charge. The AN69ST membrane was developed on the basis of an AN69 membrane marketed in France in 1969. Due to the strong negative charge of AN69 membrane, there are problems such as inducing bradykinin production and adsorbing drugs, such as nano. Therefore, for the AN69ST membrane, a surface treatment was performed to neutralize the negative charge of the membrane surface. To generate AN69ST films, the AN69 films were surface treated with biocompatible PEI, which generated weaker negative charges [37]. In the clinical setting, heparin priming is usually performed in which the membrane surface is coated with heparin; therefore, the negative charge was attenuated by adding a positive charge to the membrane. These prospective studies suggest that the high ultrafiltration volume of CVVH may improve ICU survival in critically ill septic patients [38,39].

In this study, we demonstrated that AN69ST membrane adsorbed CVVH in critically ill patients with severe sepsis and significantly decreased inflammatory parameters, including CRP, PCT, WBC, as well as secretion of inflammatory factors including IL-6 and TNF-α. IL-6 and TNF-α are classically pro-inflammatory factors that can initiate an inflammatory cascade, causing systemic waterfall-like spread of inflammation. Whereas phase protein CRP and soluble protein PCT and WBC in response to acute inflammation can respond to active progression of inflammation, treatment as well as prognosis of patients [40,41]. The results presented in our study suggest that AN69ST membrane aspiration coupled with CVVH in severe sepsis patients can significantly reduce the inflammatory indexes by inhibiting T cells to release a large number of inflammatory factors, which in turn can inhibit their secretion. Further analysis of the effect of AN69ST membrane aspiration coupled with CVVH on the liver and kidney function indicators of severe sepsis patients showed that the adsorption of AN69ST membrane to CVVH in severe sepsis patients could significantly improve the liver and kidney function indicators of severe sepsis patients, effectively improve the tissue organ perfusion, and then alleviate the tissue damage of important organs such as liver and kidney. We also analyzed the length of hospital stay, MV, and medication use in our study and found that membrane aspiration with AN69ST coupled with CVVH in critically ill patients with sepsis significantly reduced the length of hospital stay, MV, and medication use. It shows that on the one hand, thymic method can reduce the side effects of antibiotics and other drugs and long-term MV on the body, and on the other hand, the reduction of medication, hospital stay, and MV time actually reduces the economic burden of patients and their families, which is conducive to improving pharmacoeconomic indicators. Finally, we observed the effect of AN69ST membrane adsorption continuous venous hemofiltration on severe sepsis patients by analyzing APACE Ⅱ score and 30-day mortality. The results showed that the APACE Ⅱ score of patients in the experimental group was improved more significantly, and the mortality of patients with severe sepsis treated with AN69ST membrane adsorption continuous venous hemofiltration was significantly reduced 30 days after treatment. It is suggested that AN69ST membrane adsorption continuous venous hemofiltration can effectively improve the prognosis of patients with severe sepsis.

## 5 Conclusion

In this study, we confirmed that AN69ST membrane adsorption combined with CVVH in severe sepsis patients could inhibit inflammatory factors, downregulate the expression of inflammatory indicators, improve immune function, and then significantly reduce the injury of important organs *in vivo* and shorten the hospitalization time.

## References

[1] Cecconi M, Evans L, Levy M, Rhodes A. Sepsis and septic shock. Lancet. 2018;392:75–87. 10.1016/S0140-6736(18)30696-2. 29937192

[2] Rivera A, Siracusa MC, Yap GS, Gause WC. Innate cell communication kick-starts pathogen-specific immunity. Nat Immunol. 2016;17:356–63. 10.1038/ni.3375. PMC4949486 27002843

[3] Mai SHC, Sharma N, Kwong AC, Dwivedi DJ, Khan M, Grin PM, et al. Body temperature And mouse scoring systems as surrogate markers of death in cecal ligation and puncture sepsis. Intensive Care Med Exp. 2018;6(1):20. 10.1186/s40635-018-0184-3. PMC6063809 30054760

[4] Henning DJ, Puskarich MA, Self WH, Howell MD, Donnino MW, Yealy DM, et al. An emergency department validation of the SEP-3 sepsis and septic shock definitions and comparison with 1992 consensus definitions. Ann Emerg Med. 2017;70(4):544–52.e5. 10.1016/j.annemergmed.2017.01.008. PMC5792164 28262318

[5] van der Poll T, van de Veerdonk FL, Scicluna BP, Netea MG. The immunopathology of sepsis and potential therapeutic targets. Nat Rev Immunol. 2017;17(7):407–20. 10.1038/nri.2017.36. 28436424

[6] Matthay MA, Zemans RL, Zimmerman GA, Arabi YM, Beitler JR, Mercat A, et al. Acute respiratory distress syndrome. Nat Rev Dis Primers. 2019;5(1):18. 10.1038/s41572-019-0069-0. PMC6709677 30872586

[7] Peerapornratana S, Manrique-Caballero CL, Gómez H, Kellum JA. Acute kidney injury from sepsis: current concepts, epidemiology, pathophysiology, prevention and treatment. Kidney Int. 2019;96:1083–99. 10.1016/j.kint.2019.05.026. PMC6920048 31443997

[8] Kellum JA, Prowle JR. Paradigms of acute kidney injury in the intensive care setting. Nat Rev Nephrol. 2018;14:217. 10.1038/nrneph.2017.184. 29355173

[9] Drosatos K, Lymperopoulos A, Kennel PJ, Pollak N, Schulze PC, Goldberg IJ. Pathophysiology of sepsis-related cardiac dysfunction: driven by inflammation, energy mismanagement, or both? Curr Heart Fail Rep. 2015;12(2):130–40. 10.1007/s11897-014-0247-z. PMC4474734 25475180

[10] Widmann CN, Heneka MT. Long-term cerebral consequences of sepsis. Lancet Neurol. 2014;13:630–6. 10.1016/S1474-4422(14)70017-1 24849863

[11] Hirasawa H, Oda S, Nakamura M, Watanabe E, Shiga H, Matsuda K. Continuous hemodiafiltration with a cytokine adsorbing hemofilter for sepsis. Blood Purif. 2012;34(2):164–70. 10.1159/000342379. 23095416

[12] Servillo G, Vargas M, Pastore A, Procino A, Iannuzzi M, Capuano A, et al. Immunomodulatory effect of continuous venovenous hemofiltration during sepsis: preliminary data. Biomed Res Int. 2013;2013:108951. 10.1155/2013/108951. PMC3736510 23971020

[13] Heering P, Morgera S, Schmitz FJ, Schmitz G, Willers R, Schultheiss HP, et al. Cytokine removal and cardiovascular hemodynamics in septic patients with continuous venovenous hemofiltration. Intensive Care Med. 1997 Mar;23(3):288–96. 10.1007/s001340050330. 9083231

[14] Clark E, Molnar AO, Joannes-Boyau O, Honoré PM, Sikora L, Bagshaw SM. High-volume hemofiltration for septic acute kidney injury: a systematic review and meta-analysis. Crit Care. 2014 Jan;18(1):R7. 10.1186/cc13184. PMC4057068 24398168

[15] Park JT, Lee H, Kee YK, Park S, Oh HJ, Han SH, et al. High-dose versus conventional-dose continuous venovenous hemodiafiltration and patient and kidney survival and cytokine removal in sepsis-associated acute kidney injury: a randomized controlled trial. Am J Kidney Dis. 2016 Oct;68(4):599–608. 10.1053/j.ajkd.2016.02.049. 27084247

[16] Cole L, Bellomo R, Davenport P, Tipping P, Ronco C. Cytokine removal during continuous renal replacement therapy: an exvivo comparison of convection and diffusion. Int J Artif Organs. 2004 May;27(5):388–97. 10.1177/039139880402700507. 15202816

[17] Nakamura T, Moriyama K, Shimomura Y, Kato Y, Kuriyama N, Hara Y. Adsorption kinetics of high mobility group box 1 protein in a polyacrylonitrile hemofiltration membrane. Ther Apher Dial. 2021 Feb;25(1):66–72. 10.1111/1744-9987.13489. 32216030

[18] Goldfarb S, Golper TA. Proinflammatory cytokines and hemofiltration membranes. J Am Soc Nephrol. 1994 Aug;5(2):228–32. 10.1681/ASN.V52228. 7994003

[19] Yumoto M, Nishida O, Moriyama K, Shimomura Y, Nakamura T, Kuriyama N, et al. In vitro evaluation of high mobility group box 1 protein removal with various membranes for continuous hemofiltration. Ther Apher Dial. 2011;15(4):385–93. 10.1111/j.1744-9987.2011.00971.x 21884474

[20] Hattori N, Oda S. Cytokine-adsorbing hemofilter: old but new modality for septic acute kidney injury. Ren Replace Ther. 2016;2:1–8.

[21] Shiga H, Hirasawa H, Nishida O, Oda S, Nakamura M, Mashiko K, et al. Continuous hemodiafiltration with a cytokine-adsorbing hemofilter in patients with septic shock: a preliminary report. Blood Purif. 2014;38(3–4):211–8. 10.1159/000369377. 25531978

[22] Thomas M, Moriyama K, Ledebo I. AN69: evolution of the world’s first high permeability membrane. Contrib Nephrol. 2011;173:119–29. 10.1159/000328961. 21865784

[23] Nishida O, Nakamura T, Kuriyama N, Hara Y, Yumoto M, Shimomura Y, et al. Sustained high-efficiency daily diafiltration using a mediator-adsorbing membrane (SHEDD-fA) in the treatment of patients with severe sepsis. Contrib Nephrol. 2011;173:172–81. 10.1159/000329057. 21865790

[24] Honore PM, Jacobs R, Joannes-Boyau O, De Regt J, De Waele E, van Gorp V, et al. Newly designed CRRT membranes for sepsis and SIRS—a pragmatic approach for bedside intensivists summarizing the more recent advances: a systematic structured review. ASAIO J. 2013 Mar-Apr;59(2):99–106. 10.1097/MAT.0b013e3182816a75. 23438770

[25] Shiga H, Hirasawa H, Nishida O, Oda S, Nakamura M, Mashiko K, et al. Continuous hemodiafiltration with a cytokine-adsorbing hemofilter in patients with septic shock: a preliminary report. Blood Purif. 2014;38(3–4):211–8. 10.1159/000369377. 25531978

[26] Hattori N, Oda S. Cytokine-adsorbing hemofilter: old but new modality for septic acute kidney injury. Ren Replace Ther. 2016;41:2. 10.1186/s41100-016-0051-1.

[27] Arimura T, Abe M, Shiga H, Katayama H, Kaizu K, Oda S. Clinical study of blood purification therapy in critical care in Japan: results from the survey research of the Japan Society for Blood Purification in Critical Care in 2013. J Artif Organs. 2017 Sep;20(3):244–51. 10.1007/s10047-017-0968-3. 28600615

[28] Pei F, Guan X, Wu J. Thymosin alpha 1 treatment for patients with sepsis. Expert Opin Biol Ther. 2018 Jul;18(Suppl 1):71–6. 10.1080/14712598.2018.1484104. 30063866

[29] Liu Y, Pan Y, Hu Z, Wu M, Wang C, Feng Z, et al. Thymosin alpha 1 reduces the mortality of severe coronavirus disease 2019 by restoration of lymphocytopenia and reversion of exhausted T cells. Clin Infect Dis. 2020 Nov 19;71(16):2150–7. 10.1093/cid/ciaa630. PMC7314217 32442287

[30] Mirouse A, Vigneron C, Llitjos JF, Chiche JD, Mira JP, Mokart D, et al. Sepsis and cancer: an interplay of friends and foes. Am J Respir Crit Care Med. 2020 Dec 15;202(12):1625–35. 10.1164/rccm.202004-1116TR. 32813980

[31] Romagnoli S, Ricci Z, Ronco C. CRRT for sepsis-induced acute kidney injury. Curr Opin Crit Care. 2018 Dec;24(6):483–92. 10.1097/MCC.0000000000000544. 30239411

[32] Moriyama K, Nishida O. Targeting cytokines, pathogen-associated molecular patterns, and damage-associated molecular patterns in sepsis via blood purification. Int J Mol Sci. 2021 Aug;22(16):8882. 10.3390/ijms22168882. PMC8396222 34445610

[33] Bellomo R, Cole L, Reeves J, Silvester W. Who should manage CRRT in the ICU? The intensivist’s viewpoint. Am J Kidney Dis. 1997;30(5 Suppl 4):S109–S111. 10.1016/s0272-6386(97)90552-7 9372989

[34] De Vriese AS, Colardyn FA, Philippé JJ, Vanholder RC, De Sutter JH, Lameire NH. Cytokine removal during continuous hemofiltration in septic patients. J Am Soc Nephrol. 1999 Apr;10(4):846–53. 10.1681/ASN.V104846. 10203370

[35] VA/NIH Acute Renal Failure Trial Network, Palevsky PM, Zhang JH, O’Connor TZ, Chertow GM, Crowley ST, et al. Intensity of renal support in critically ill patients with acute kidney injury. N Engl J Med. 2008 Jul 3;359(1):7–20. 10.1056/NEJMoa0802639. PMC2574780 18492867

[36] RENAL Replacement Therapy Study Investigators, Bellomo R, Cass A, Cole L, Finfer S, Gallagher M, et al. Intensity of continuous renal-replacement therapy in critically ill patients. N Engl J Med. 2009 Oct 22;361(17):1627–38. 10.1056/NEJMoa0902413. 19846848

[37] Lee KH, Ou SM, Tsai MT, Tseng WC, Yang CY, Lin YP, et al. AN69 filter membranes with high ultrafiltration rates during continuous venovenous hemofiltration reduce mortality in patients with sepsis-induced multiorgan dysfunction syndrome. Membranes (Basel). 2021 Oct;11(11):837. 10.3390/membranes11110837. PMC8618352 34832066

[38] Ronco C, Bellomo R, Homel P, Brendolan A, Dan M, Piccinni P, et al. Effects of different doses in continuous veno-venous haemofiltration on outcomes of acute renal failure: a prospective randomised trial. Lancet. 2000 Jul;356(9223):26–30. 10.1016/S0140-6736(00)02430-2. 10892761

[39] Ratanarat R, Brendolan A, Piccinni P, Dan M, Salvatori G, Ricci Z, et al. Pulse high-volume haemofiltration for treatment of severe sepsis: effects on hemodynamics and survival. Crit Care. 2005 Aug;9(4):R294–302. 10.1186/cc3529. PMC1269433 16137340

[40] Teixeira C, Rosa RG. Post-intensive care outpatient clinic: is it feasible and effective? A literature review. Rev Bras Ter Intensiva. 2018 Mar;30(1):98–111. 10.5935/0103-507x.20180016. PMC5885237 29742221

[41] Lin HY. The severe COVID-19: a sepsis induced by viral infection? And its immunomodulatory therapy. Chin J Traumatol. 2020 Aug;23(4):190–5. 10.1016/j.cjtee.2020.06.002. PMC7451584 32690231

